# Interaction of the nitrogen-containing carbon backbone polymers with essential α-amino acids

**DOI:** 10.55730/1300-0527.3458

**Published:** 2022-06-13

**Authors:** Andrey V. SOROKIN, Maria S. LAVLINSKAYA

**Affiliations:** 1 Laboratory of Metagenomics and Food Biotechnologies, Voronezh State University of Engineering Technologies, Voronezh; 2 Bioresource Potential of the Seaside Territory Laboratory, Sevastopol State University, Sevastopol

**Keywords:** Free radical polymerization, poly(*N*-vinylamides), poly(*N*-vinylazoles), copolymers, amino acids, conjugation

## Abstract

The aim of this work is to research the interactions of water-soluble nitrogen-containing copolymers with essential amino acids in aqueous media. For this, poly(*N*-vinylformamide-*co*-*N*-vinylimidazole) and poly(*N*-vinylcaprolactam-*co*-*N*-vinylimidazole) random copolymers were synthesized by free radical polymerization. The products obtained are characterized by GPC, DLS, and FTIR. The copolymers have a narrow molecular weight distribution and low dispersity. The interactions of the obtained copolymers with histidine, proline, arginine, leucine, phenylalanine, and methionine were researched by UV spectroscopy, FTIR, and TEM. It was found that conjugation of the copolymers with amino acids correlates with the copolymer composition and hydrodynamic radius *R_h_*, and depends on the pH of the medium and amino acid structures. It is shown that chloride anion presence in the polymer-amino acid-water systems affects the mechanism of their interactions. The research shows that the synthesized copolymers can be used for the creation of effective eco-friendly amino acid extraction systems or matrices for enzyme immobilization.

## 1. Introduction

Amino acids are biological compounds that are essential for human and animal organisms. They are precursors for the synthesis of proteins and other biologically active substances, as well as take part in metabolism regulation. A great amount of amino acids is industrially produced by microbial synthesis or by protein hydrolysis [1]. Every amino acid production includes amino acid extraction and concentration from the raw materials, and nowadays these processes are characterized by some drawbacks, such as low efficiency, long-time duration, and expensive equipment [1]. Hence, development of the new, eco-friendly and effective ways of amino acid extraction and concentration is a considerable goal for the researchers. Liquid extraction based on water-soluble polymers replacing toxic and flammable organic solvents is a cheap and express concentration method providing the process effectiveness and corresponding to the modern “green” trends in chemistry [2]. Effective extraction in such systems is achieved due to nonspecific and specific interactions between amino acids and polymers. The last type is observed for polymers whose macrochains contain functional groups and are characterized by a high complexing ability [3]. For example, poly(*N*-vinylamides), poly(*N*-vinylazoles), polyacrylates, and their copolymers are effective extractants for inorganic [4–8] and organic substances [9–15]. Also, some poly(*N*-vinylamides), such as poly(*N*-vinylformamide) and poly(*N*-vinylcaprolactam), is characterized by low toxicity and biocompatibility, which allows them to be used in biomedicine and biotechnologies [2, 4, 5, 11, 16–20]. Poly(*N*-vinylimidazole) and its derivatives are applied in pharmaceutics and cosmetology [16, 18, 21–25].

Moreover, as mentioned above, amino acids are protein building blocks. Some proteins, i.e. enzymes, have a significant impact on the biotechnological industry. However, there are some restrictions on commercial enzyme uses, such as low stability and half-life time in the aqueous solutions [26–28]. The enzyme formulations immobilized onto different polymers can enhance industry applications of the ones. Therefore, when creating new enzyme immobilized formulations, it is necessary to know enzyme interactions with the polymer matrix and reveal the driving forces and mechanism of the process to predict the properties of the obtaining formulations. However, a significant part of the industrially used enzymes are expensive, so to understand the interaction mechanism of the ones with polymers it is more reasonable to investigate the interaction of model unlinked amino acids with the polymer matrix [26–28].

However, today there is no systematic research on the interaction mechanism of the water-soluble poly(*N*-vinylamides) and poly(*N*-vinylazoles) with amino acids, despite the fact that the study of polymer-amino acid interaction can enhance polymer application in the field of amino acid concentration and immobilized enzyme production.

In this connection, the goal of this work is the study of complexation in aqueous systems of carbon backbone nitrogen-containing copolymers of *N*-vinylcaprolactam (VC), *N*-vinylimidazole (VI), and *N*-vinylformamide (VF) with α-amino acids histidine, proline, phenylalanine, leucine, arginine, and methionine.

## 2. Materials and methods

### 2.1 Materials

*N*-vinylcaprolactam (VC) with mp = 32–34 °C; bp = 92–93 °C/1 mm Hg, *N*-vinylimidazole (VI) with bp = 78–79 °C/11 mm Hg; n_D_^20^ 1.5338, *N*-vinylformamide (VF) with bp = 78–79 °C/10 mm Hg; n_D_^20^ 1.5330, all Acros Organic, USA, were applied in the work and were purified immediately before use by vacuum distillation or by recrystallization from hexane for VC. Azobis(isobutyronitrile) (AIBN), Acros Organics, USA, recrystallized from ethanol with mp = 102–103 °C was applied as an initiator. Histidine (His), methionine (Met), arginine (Arg), leucine (Leu), phenylalanine (Phe) and proline (Pro) (Aldrich, Germany) was chemically pure grade and used without any purification. Propanol-2 and hexane, all Aldrich, Germany, were used as solvents. Absolute ethanol and distilled water were used for intrinsic viscosity and DLS measurements.

### 2.2 Synthesis of the copolymers

Poly(*N*-vinylcaprolactam-*co*-*N*-vinylimidazole) copolymers denoted as P(VC-VI) and poly(*N*-vinylformamide-*co*-*N*-vinylimidazole) copolymers denoted as P(VF-VI) with different mole compositions in the range 0.1–0.9:0.9–0.1 mol frac were produced by free radical polymerization as described in [2]. The polymerization mixtures were degassed by 3 freeze-pump-thaw cycles with liquid nitrogen use. The copolymers obtained were fractionated by precipitation in hexane-ethanol mixtures (95/5–85/15 v/v). The fractions with the highest yield for all copolymers were collected, reprecipitated, dried in a vacuum oven to a constant weight, and used for further research.

### 2.3. Instrumental section

#### 2.3.1. Determining the copolymer compositions

The FTIR spectra were recorded in the range of 4400–400 cm^−1^ with a Bruker Vertex 70 Fourier transform spectrophotometer (Bruker Optics, Germany) in the ATR mode. The composition of the copolymers was calculated from the ratio of the areas of absorption bands corresponding to stretching vibrations of the >C=O groups (the range near 1636–1647 cm^−1^) of VF and VC of the absorption band at 1516 cm^−1^ belonging to the azole rings [29, 30]. The samples were in a powder or wet form.

The ^1^H NMR spectra of the synthesized copolymers were recorded with AVANCE II 600 spectrometer (Bruker Corporation, Germany) using, in 0.5 w/v D_2_O solutions with TMS internal standard, operation frequency was 600 MHz.

#### 2.3.2. Determining molecular weight and intrinsic viscosity

Molecular weight determination was carried out by gel permeation chromatography (GPC) as described in [2].

The intrinsic viscosity [η] values were calculated from the viscosity data obtained in ethanol at 20 ± 0.2 °C with an Ubbelohde viscometer.

#### 2.3.2. Dynamic light scattering

Dynamic light scattering (DLS) measurements were performed using a PhotoCor Complex spectrometer (PhotoCor Instruments, Russia) equipped with a He–Ne laser as a light source (λ = 633 nm). Measurements for determining the hydrodynamic radius *R_h_* were performed in dilute solutions at 25 °C within scattering angles of 90**°**. Sample solutions were filtered through Millipore membrane filters with rated pore sizes of 0.45 μm.

#### 2.3.3. Transmission electron microscopy

Transmission electron microscopy (TEM) was performed with a Libra 120 Carl Zeiss electron microscope, Germany, in bright field mode. The samples were prepared as described in [2].

### 2.4. Investigation of the complexation by UV spectroscopy and FTIR

For studies of the interaction between copolymers (*c* = 0.5% w/v) and amino acids in water solutions, their characteristic absorption maxima **λ***_max_* were recorded with UV-Vis spectrometer Shimadzu UV-1800 (Shimadzu Scientific Instruments, Japan). Then, after the addition of the amino acid solutions in the 0.2–3.0 **×** 10^−4^ mol L^−1^ concentration range, the shifts of **λ***_max_* values were registered at 192 nm for VC and VF links, and at 312 nm for VI units. For specification of the interaction mechanism, the same solutions were analyzed by FTIR.

## 3. Results and discussion

### 3.1. Synthesis and characterization of the copolymers

**Figure 1:**
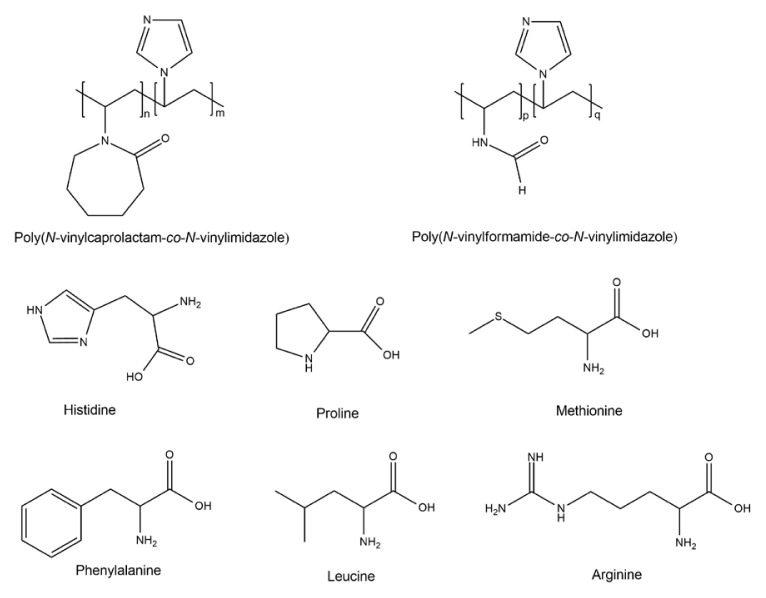
The structures of the research objects.

Poly(*N*-vinylcaprolactam-*co*-*N*-vinylimidazole), denoted as P(VC-VI), and poly(*N*-vinylformamide-*co*-*N*-vinylimidazole), denoted as P(VF-VI), with different compositions were obtained to research their complexing ability towards essential amino acids histidine, proline, arginine, leucine, phenylalanine, and methionine (Figure 1). The reactions were performed in propanol-2 solutions with an AIBN initiator. According to the previously published studies, the copolymers obtained by free radical polymerization have a random comonomer link distribution [2]. The absence of the characteristic low intensive absorption bands near 1410 cm^−1^ corresponding to δ H_2_C=CH- in FTIR spectra copolymers obtained confirms that the polymerization proceeds by opening the double bonds of the monomer vinyl group [29].

**Figure 2:**
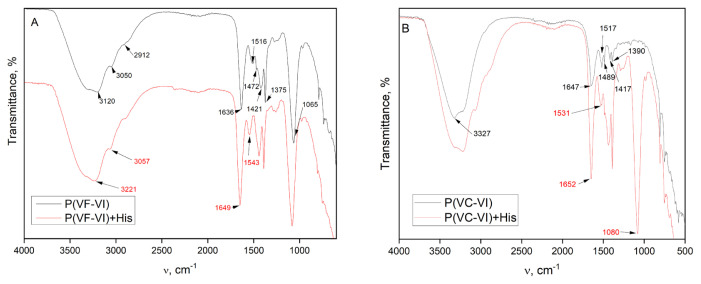
The FTIR spectra.

The structures of the obtained copolymers were confirmed by FTIR (Figure 2). The typical spectrum of P(VF-VI) contains the following characteristic intensive absorption bands: at 1065 cm^−1^ corresponding to C-H vibrations of imidazole rings; at 1375 cm^−1^ representing main polymer backbone vibrations; at 1421 cm^−1^ ascribed to asymmetric bending vibrations of the C-N bonds in amides, at 1472 cm^−1^ for ripple stretching vibrations of the azole rings; at 1516 cm^−1^ attributed to stretching vibrations of the C=N bonds in the azole rings; at 1636 cm^−1^ for stretching vibrations of C=O, at 2912 cm^−1^ and 3050 cm^−1^ representing C-H stretching vibrations of the main backbones and azole rings, respectively; and a broad band at 3120 cm^−1^ for associated NH- and OH- group vibrations [29, 30].

The typical FTIR spectrum of P(VC-VI) is similar to the one above and contains the following characteristic absorption bands (Figure 2B): at 1390 cm^−1^ representing main polymer backbone vibrations; at 1417 cm^−1^ ascribed to asymmetric bending vibrations of the C-N bonds in amides; at 1489 cm^−1^ for ripple stretching vibrations of the azole rings; at 1517 cm^−1^ attributed to stretching vibrations of the C=N bonds in the azole rings; at 1647 cm^−1^ for stretching vibrations of C=O; at 3327 cm^−1^ corresponding to associated OH- group vibrations [29, 30].

**Figure 3:**
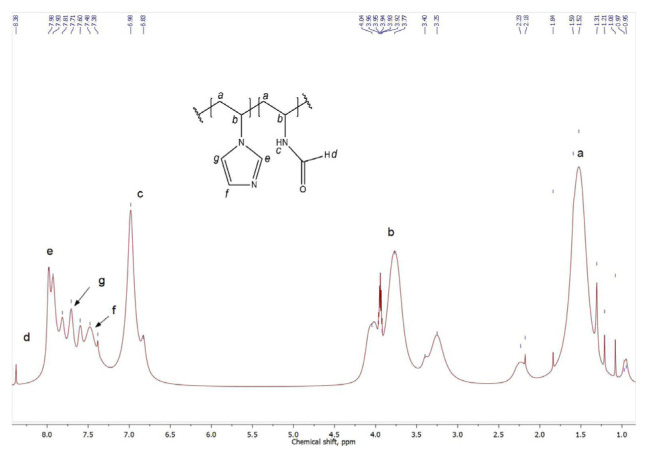
The ^1^H NMR spectrum of the synthesized P(VF-VI) copolymer.

The structures of the synthesized copolymers are also confirmed by ^1^H NMR method. The typical spectrum of P(VF-VI) copolymer is represented in Figure 3, and it contains the signal descriptions. The ^1^H NMR spectrum of P(VC-VI) copolymer is typical for such polymers and contains the signals at δ = 1.72–3.45 ppm and 7.00–7.69 ppm corresponding to protons of amide and azole cycles, respectively. Copolymer compositions were calculated from FTIR and ^1^H NMR data (Table 1).

**Figure 4:**
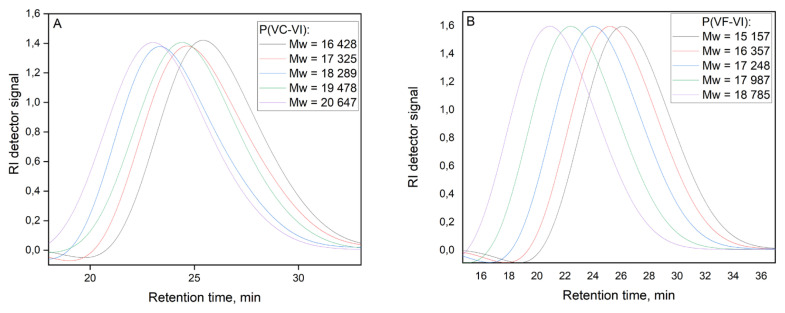
The GPC curves of the synthesized copolymers.

Molecular weights of the copolymers after fractionation were calculated using GPC data. For analysis, the main fractions (about 70%–85% w of obtained copolymers) were selected. The GPC curves (Figure 4) are similar for all copolymers and demonstrate unimodal distribution. As can be seen, with the decrease of VI links in the copolymers, *M_N_* and *M_W_* values grow in both systems. These results are in good agreement with the existing research data: it is well known that *N*-vinylimidazole is characterized by the degradation chain transfer due to the formation of resonant forms of the growing macroradical [31], and this is the reason for the molecular weight decreasing for copolymers enriched in VI links. Moreover, the dispersity is almost identical for all copolymers and points to relatively narrow molecular weight distribution in all composition ranges. The choice of the molecular weight range of the synthesized copolymers is since polymers with high molecular weights are toxic to a living organism, and copolymers with a lower molecular mass have almost no complexing ability.

**Figure 5:**
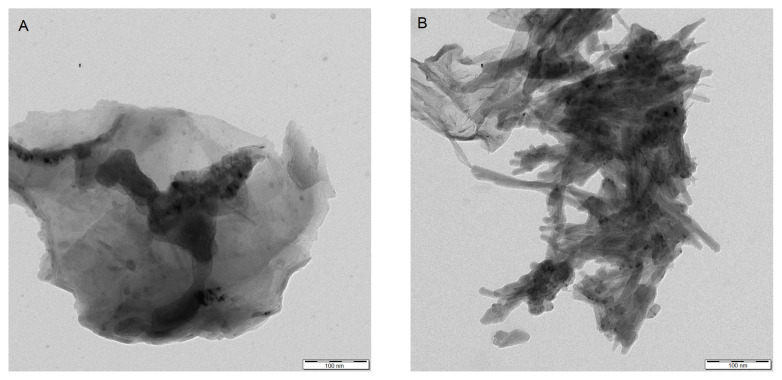
The TEM images of the P(VF-VI) copolymer particles with 0.26 VF mol frac (A) and their conjugate with histidine (B).

The *R_h_* values were determined for all copolymers obtained in 0.5% w/v water solutions by the DLS method. It was established that *R_h_* values of the copolymers depend on their composition and increase with VI content growth. Also, the *R_h_* values obtained show that copolymers in 0.5% w/v solutions are associated into multichain aggregates [4]. The DLS results obtained correlates with the TEM data. Figure 5 represents the TEM image of the synthesized copolymers. As can be seen, the copolymer particles have a nondefined form and core-shell-like architecture with a denser core. Moreover, this is typical for self-assembled (co)polymers [21, 22].

These results also correlate with intrinsic viscosity data. The intrinsic viscosity values [η] for all synthesized copolymers were determined in ethanol at 20 °C. It was found that [η] meanings for P(VC-VI) copolymers are in the range 0.19–0.51 dL g^−1^, and increase with VI content growth. For P(VF-VI) copolymers, [η] values also increase with VI content growing in the range 0.15–0.50 dL g^−1^. This tendency correlates with the propensity of VI-based (co)polymers to self-association [22].

The dependence of hydrodynamic radius *R_h_* on intrinsic viscosity [η] of the spherical particles can be expressed by the following equation [32]:


(1) $$R_{h}={\left(\frac{3[\eta ]M}{10\pi N}\right)}^{1/3},$$

where *M* is polymer molecular weight, *N* is Avogadro’s number. As can be seen from the TEM data, the copolymer particles in solutions have deviated spherical form and are associated; however, the calculated *R_h_^calc^* values (Table 1) are in good agreement with the experimental data. A better correlation of the *R_h_* and *R_h_^calc^* is observed for P(VF-VI) copolymers, which are characterized by less self-association compared to P(VC-VI) copolymers. Also, the main tendency described in Eq. 1 is kept: *R_h_* values increase with the growth of the molecular weight and intrinsic viscosity of the copolymers.

Therefore, copolymers P(VC-VI) and P(VF-VI) with different compositions were successfully synthesized by free radical solution polymerization. It was established that the molecular weight of the copolymers obtained decreases with the VI content growth; however, their intrinsic viscosity and hydrodynamic radius increase with VI content growth.

### 3.2. Researching the complexing ability of the synthesized copolymers

Molecular spectroscopy methods are a cheap and simple way of investigating the interaction in solutions. To research the complexation of the synthesized copolymers with amino acids in an aqueous medium UV-spectroscopy and FTIR methods were used. It is known that the stability of the resulting complex can be indirectly judged by the shifts of the characteristic absorption maxima (*λ_max_*) in UV spectra [2]. Thus, the larger the shift value *λ_max_* in the complexing-ligand mixture, the more stable is the complex formed between them.

**Figure 6:**
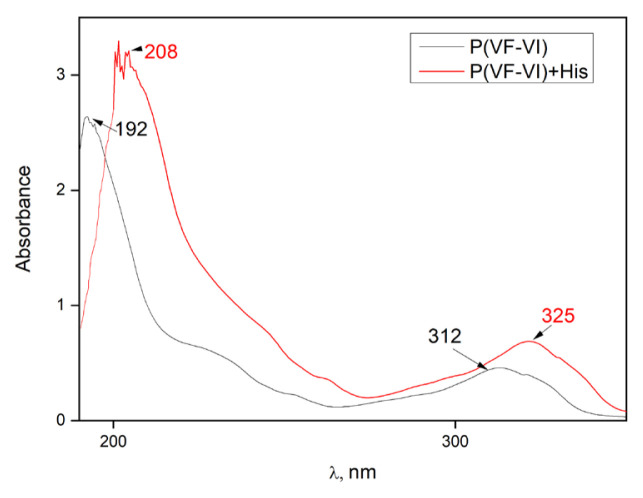
The UV spectrum of P(VF-VI) copolymer and its mixture with histidine (pH = 5.5 ± 0.2).

**Figure 7:**
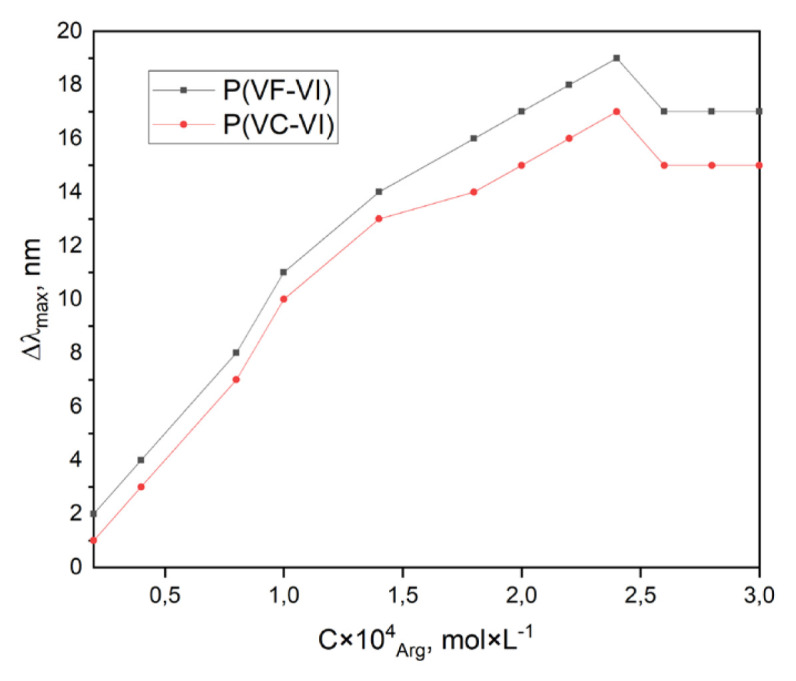
The dependence of the absorption maximum shift on the histidine concentration.

The UV spectrum of the P(VF-VI) in distilled water (pH = 5.5 ± 0.2) is represented in Figure 6 and contains characteristic absorption maxima at 192 nm corresponding to C=O groups, and at 312 nm for the imidazole ring light absorption. The P(VC-VI) UV spectrum is the same due to the identical chromophore groups. The maximum at 192 nm is more intensive, thus it was chosen for further research as *λ_max_*. The absorption maxima shifts are observed in the UV spectrum of the P(VF-VI) and blend with amino acid (Figure 6, Table 2). Studying the dependence of Δ*λ_max_* of mixtures on the histidine concentration (Figure 7) shows that the least stable complex is formed in the P(VC-VI) with 0.89 VC mol frac-His system, and the strongest one is formed by the P(VF-VI) with 0.26 VF mol frac-His at a 2.4 × 10^−4^ mol L^−−1^ histidine concentration. The decreasing of the Δ*λ_max_* values at a concentration higher than 2.4 × 10^−4^ mol L^−1^ is probably due to amino acid self-assembling [33, 34].

The experimental data for other amino acids correlate with the ones obtained for histidine and are represented in Table 2. As can be seen from the data presented, the maximum Δλ_max_ value is observed for arginine, and the minimum one is for leucine, and decreases in the order Arg>His>Pro>Phe>Met>Leu. These results obtained correlate with the amino acid polarity (Table 2) which is due to their structure [1]. Due to the structure of the amino acids researched and their molecular functional groups, it is clear that arginine and histidine have a higher affinity to the hydrophilic copolymers synthesized compared to other amino acids, especially hydrophobic leucine and methionine.

The copolymer composition and hydrodynamic radius values also affect the interaction with amino acids. As can be seen from Table 2, the Δλ_max_ values rise with VI content growth in the copolymers. Moreover, with the increase of the *R_h_* values, Δλ_max_ also rises. Therefore, we can conclude that VI links have more affinity to the amino acids compared to amide links and promote more sterical availability for interactions with amino acids.

Due to fact that the form of the amino acid existence in the solution depends on the medium pH, this effect was studied. In an acidic medium, an amino acid cation with a protonated amino group dominates in the aqueous solution, while at pH > 7, the majority of the molecules contain a dissociated carboxyl group, i.e. are in the anion form. In addition, usually in the solution of amino acids, there is an internal salt called zwitterion or betaine, which is stable in a wide pH range [1].

It is well known that the pH of the medium affects amino acid structure. Therefore, the research on the interactions between amino acids and synthesized copolymers was performed at some pH values. From the UV spectroscopy data, it is established that the largest absorption maximum shifts are observed in media with an acid reaction, and the minimal ones are in an alkaline medium (Table 2). This behavior is explained by the enhancement of the electrostatic interaction between differently charged fragments of polymers and amino acids with the participation of water molecules. Complexation between polymers and amino acids occurs by the formation of hydrogen bonds between the >C=O groups and azole cycles of the copolymers and -COOH, -COO^−^ and ^+^NH_3_ groups of amino acids. The formation of hydrogen bonds between the >C=O groups and azole rings of the lateral substituent of the copolymers with amino acids in water is indicated by the data of FTIR spectroscopy.

For example, in the P(VF-VI)-histidine-water system, the stretching vibration band ν_C=O_ of VF links is at 1649 cm^−1^, and it is observed at 1636 cm^−1^ ν_C=O_ for the P(VF-VI) water system (Figure 2A). It is considered [2] that complexation with the nitrogen atom of the VC cycles is difficult due to the influence of the steric factor from the main chain. The vibration band ν_C=N_ of the azole cycles shifts from 1515 cm^−1^ to 1543 cm^−1^, which indicates the possible formation of H-bonds between the copolymers and amino acids. The same case is observed for the P(VC-VI)-water-histidine-system: the ν_C=O_ band of the VC links shifts from 1647 cm^−1^ to 1652 cm^−1^, ν_C =N_ band of the azole cycle shifts from 1517 cm^−1^ to 1531 cm^−1^ (Figure 2B). The interaction of the functional groups of the polymer with bipolar ions as well as with the undissociated amino acid by a similar mechanism is possible. The possible dipole-dipole, ion-ion, ion-dipole interactions, as well as hydrophobic interactions of the methylene groups of the polymer backbone and the side substituent with the corresponding amino acid fragments should not be excluded.

The TEM image of the P(VF-VI) conjugate is represented in Figure 5B. As can be seen, the particle size increases and the form changes to angular after interactions with histidine, while the core-shell-like particle architecture is preserved. The more angular form can be attributed to histidine that can form needle-like crystals [35].

To clarify the mechanism of the interaction of polymers with amino acids in aqueous solutions, the chloride anion binding ^+^NH_3_ group of amino acids was added to the polymer-amino acid mixture. In the FTIR spectra of these mixtures, there are practically no changes in the characteristic absorption bands corresponding to the amino groups of the amino acids. At the same time, for all systems containing His, Pro, and Arg the shift of the characteristic vibration bands remains. This indicates the presence of interaction between heterocycles of polymers and amino acids. The same data correspond to the results of determining the number of unbound amino acids in the P(VF-VI)-amino acid-Cl^−^ systems (Table 3). To determine them, the polymer complex was removed from the aqueous solution, and the residual concentration of amino acids was determined spectrophotometrically. The presence of interaction between the studied polymers and amino acids makes it possible to use synthesized materials for the extraction of amino acids.

**Figure 8:**
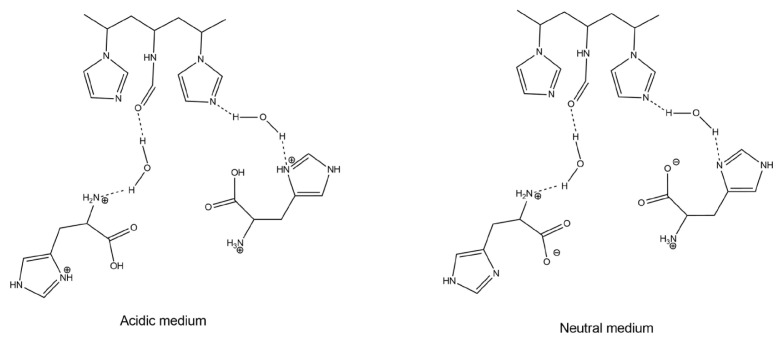
The possible P(VF-VI)-His conjugate structures.

Based on the experimental data, we can conclude regarding the structure of the associates (Figure 8).

## 4. Conclusion

Thus, water-soluble copolymers poly(*N*-vinylformamide-*co*-*N*-vinylimidazole) and poly(*N*-vinylcaprolactam-*co*-*N*-vinylimidazole) were synthesized by free radical polymerization. The copolymers obtained are characterized by GPC, DLS, and FTIR. The fractionated copolymers have a narrow molecular weight distribution and low PDI values. The interaction of the copolymers obtained with histidine, proline, arginine, leucine, phenylalanine, and methionine was researched by UV spectroscopy, FTIR, and TEM. It was found that interaction of the copolymers with amino acid decreases in the order Arg>His>Pro>Phe>Met>Leu. Researching histidine interaction with the copolymers, it has been established that the most complete interaction occurs in an acid medium with the amino acid concentration of 2.4 × 10^−4^ mol L^−1^ by the hydrogen bonds formed between carbonyl groups and imidazole cycles of the copolymers and amino groups of the amino acid. Therefore, synthesized copolymers can be used for creating effective amino acid extraction systems.

## Figures and Tables

**Table 1:** Characterization of synthesized copolymers ([M_1_] + [M_2_] = 1 mol L^−1^, *C*_AIBN_ = 1 × 10^−2^ mol L^−1^, 65 °C, τ = 8 h).

VC/VF in monomer feed, mol frac	VC/VF in copolymer, mol frac		[η], dL g^−1^, ethanol, 20 °C	M_w_	M_n_	Dispersity	*R_h_*, nm, water	*R_h_^calc^*, nm
	FTIR	^1^H NMR						
P(VC-VI)								
0.10	0.24	0.33	0.50	16 428	12 445	1.32	261	186
0.30	0.47	0.58	0.49	17 325	12 554	1.38	224	182
0.50	0.52	0.58	0.33	18 289	12 971	1.41	196	148
0.70	0.78	0.83	0.24	19 478	14 013	1.39	171	125
0.90	0.89	0.92	0.15	20 647	15 182	1.36	157	96
P(VF-VI)								
0.10	0.26	0.36	0.51	15 157	11 749	1.29	189	175
0.30	0.41	0.49	0.42	16 357	12 486	1.31	163	162
0.50	0.53	0.56	0.33	17 248	12 590	1.37	137	143
0.70	0.73	0.77	0.29	17 987	12 667	1.42	119	135
0.90	0.88	0.91	0.19	18 785	13 418	1.40	102	105

**Table 2:** The copolymer absorption maximum shifts Δλ_max_ in the UV spectra after interaction with amino acids in aqueous solutions.

VC/VF in copolymer	Δλ_max_, nm																	
	pH = 2.5 ± 0.2						pH = 5.5 ± 0.2						pH = 9.5 ± 0.2					
	Arg	His	Pro	Phe	Met	Leu	Arg	His	Pro	Phe	Met	Leu	Arg	His	Pro	Phe	Met	Leu
Amino acid polarity	20.0	10.3	6.0	0.8	−1.5	−2.3	20.0	10.3	6.0	0.8	−1.5	−2.3	20.0	10.3	6.0	0.8	−1.5	−2.3
P(VC-VI)																		
0.24	18	17	13	10	9	6	17	14	12	9	7	5	13	11	9	7	4	2
0.47	17	16	12	9	8	5	16	13	10	8	5	5	12	10	8	6	3	2
0.52	16	16	12	9	8	4	15	13	10	7	5	4	12	10	8	6	3	1
0.78	16	15	11	8	7	4	14	12	9	5	4	2	11	9	6	5	2	1
0.89	15	14	10	7	6	3	12	11	8	5	3	1	11	9	6	5	2	1
P(VF-VI)																		
0.26	20	18	16	11	10	7	19	16	14	11	9	6	14	12	9	8	6	4
0.41	19	17	15	10	9	6	17	15	12	9	7	5	13	13	8	7	5	3
0.53	18	17	14	10	9	6	15	13	11	7	6	4	13	12	7	6	4	2
0.73	17	16	14	9	8	5	14	12	10	6	5	2	12	11	7	6	3	1
0.88	16	15	13	8	7	4	14	12	9	6	4	1	11	10	6	5	3	1

**Table 3:** Influence of chloride anion on the complex ability of the polymers.

C×10^4^_Cl−_, mol L^−1^	0	0.6	1.2	2.4	4.5	9.0
Amino acid	Unlinked amino acids, %					
Arg	31.4	34.3	37.4	41.2	44.3	46.2
His	37.6	40.2	43.6	48.1	48.9	50.6
Pro	41.8	43.7	45.0	46.6	49.3	50.8
Phe	57.4	62.2	66.4	71.5	74.9	76.9
Met	66.5	73.5	79.9	89.6	90.5	91.4
Leu	74.2	79.6	85.5	91.8	95.4	96.6
